# A machine learning approach to triaging patients with chronic obstructive pulmonary disease

**DOI:** 10.1371/journal.pone.0188532

**Published:** 2017-11-22

**Authors:** Sumanth Swaminathan, Klajdi Qirko, Ted Smith, Ethan Corcoran, Nicholas G. Wysham, Gaurav Bazaz, George Kappel, Anthony N. Gerber

**Affiliations:** 1 Revon Systems Inc, Louisville, KY, United States of America, 40014; 2 Department of Mathematics, University of Delaware, Newark, DE, United States of America, 19716; 3 Department of Pulmonology, Kaiser Permanente, Clackamas, OR, United States of America, 97015; 4 Vancouver Clinic Division of Pulmonology & Critical Care, Vancouver, WA, United States of America, 98664; 5 Washington State University School of Medicine, Spokane, WA, United States of America, 99210; 6 Department of Medicine, National Jewish Health, Denver, CO, United States of America, 80206; Ospedale S. Corona, ITALY

## Abstract

COPD patients are burdened with a daily risk of acute exacerbation and loss of control, which could be mitigated by effective, on-demand decision support tools. In this study, we present a machine learning-based strategy for early detection of exacerbations and subsequent triage. Our application uses physician opinion in a statistically and clinically comprehensive set of patient cases to train a supervised prediction algorithm. The accuracy of the model is assessed against a panel of physicians each triaging identical cases in a representative patient validation set. Our results show that algorithm accuracy and safety indicators surpass all individual pulmonologists in both identifying exacerbations and predicting the consensus triage in a 101 case validation set. The algorithm is also the top performer in sensitivity, specificity, and ppv when predicting a patient’s need for emergency care.

## Introduction

Chronic Obstructive Pulmonary Disease (COPD) is a serious long-term lung condition that progressively restricts airflow from the lungs and imposes a significant burden on patients’ daily lives. COPD includes a spectrum of pulmonary phenotypes with emphysema and chronic bronchitis being the two most prominent members. Flare-ups (or exacerbations) are a frequent trigger of physician and hospital visits that are both costly and distressing to patients. Moreover, exacerbations are associated with long term declines in lung function and health status [1, 2]. A World Health Organization report anticipates that by 2030, COPD will become the third leading cause of mortality and the seventh leading cause of morbidity worldwide [3].

Despite the recognized impact of exacerbations on morbidity, mortality, and health status, there is no standardized clinical approach to improve self-identification of COPD exacerbations by patients at home. Perhaps the most widely used system is a physician provided paper checklist or “action plan”. In these instances, patients are instructed to refer to a document when they are feeling concerned about their breathing [4, 5]. The document generally has green, yellow, and red zones, which guide patients to continue usual treatment, call a physician, or go to the emergency room if their symptoms match those designated in a particular zone [6, 7]. While the type of medical guidance offered in these checklists has demonstrated some utility in patient education [8, 9], the method of delivering that guidance through a hard-coded list lacks rigor, validation, and robustness at the level of the individual patient [10]. It is not surprising then, that urgent calls or visits to the emergency room may provide the fastest path to feedback especially during hours when a doctor’s office is closed. In fact, COPD is one of the leading chronic conditions driving potentially avoidable hospital admissions [11]. The need for novel solutions that limit the impact of exacerbations on patient health is abundantly apparent.

Both in COPD and many other chronic diseases, telemonitoring and mobile application-based tools have generated a great deal of excitement as novel, nonpharmacologic strategies to improve home-based disease management [12, 13]. In many cases, however, clinical examinations of such approaches have struggled to show statistically significant efficacy [14–16]. In COPD, one difficulty in enabling early diagnosis and potential treatment of exacerbation is the lack of a specific predictive diagnostic criteria. For example, the American Thoracic Society (ATS) defines a COPD exacerbation as, “an event in the natural course of the disease characterized by a change in the patient’s baseline dyspnea, cough, and/or sputum and beyond normal day-to-day variations, that is acute in onset and may warrant a change in regular medication in a patient with underlying COPD” [17]. This definition is highly ambiguous given the range, duration, and severity of possible COPD symptoms, which makes a definitive diagnosis of a COPD exacerbation challenging.

Compounding the issue of diagnosis is the inherent complexity in the interdependence of clinical features. For example, a rule-based system that dictates recommendations based on oxygen saturation or pulse may struggle to deliver appropriate guidance to a Gold Stage 1 COPD patient, who likely has normal baseline pulse and oxygen saturation in comparison to a Gold Stage 4 patient, who is likely to have abnormal baseline values [18]. Moreover, the myriad of patient physiologic profiles within individual Gold stages confounds efforts to create effective nested rules systems. Thus, app-based solutions that simply mimic paper-based home action flowcharts are unlikely to result in important improvements in patient outcomes. Machine-learning methods have gained considerable attention as novel strategies for capturing the interdependence of health variables when making predictions of complex health events [19–21]. In this study, we developed one such approach to provide both at-home decision support and an assessment of the possibility of a disease flare-up to COPD patients. We started by performing a detailed literature search and conducting an expert opinion review to define key patient characteristics including demographics, comorbid conditions, history, symptoms and vitals signs that are sufficiently and robustly predictive of exacerbation risk [22–31]. We used these variables to generate clinically diverse, simulated patient cases, and we asked physicians to provide their opinion on 1) the severity of the patient’s baseline health, vital signs, and current symptoms, 2) whether or not the patient was experiencing an exacerbation, and 3) the appropriate triage category for the patient. Physician labeled data sets were used to train a supervised machine-learning algorithm that predicts the likelihood that a patient is having a COPD flare-up and provides guidance on the appropriate responsive action. The algorithm feature set included a diverse mix of current and baseline health data. The model’s performance was validated by comparing its predictions to the consensus decision of a panel of physicians in an out-of-sample representative patient set. Analysis of the algorithm performance and the physician provided data showed 1) the algorithm showed exceptional performance when compared to individual pulmonologists in assessing the likelihood that a patient is experiencing an exacerbation and identifying the appropriate consensus triage, 2) the algorithm triaged in favor of the safety of the patient, when disagreeing with consensus, more often than individual physicians, and 3) the algorithm decision making was transparent and consistent when compared to participating pulmonologists.

## Methods

### Physician selection

Physician input was used to facilitate three major aspects of the algorithm development process:

1. Algorithm feature (clinical variable) selection,
2. Algorithm training data,
3. Algorithm validation data.

All participating physicians were board certified pulmonologists and/or critical care specialists from both private and academic institutions. Refer to S1 Table for the profiles of the physicians and their respective roles in this study.

### Algorithm feature selection & patient case generation

The most relevant patient symptoms, vital signs, and baseline characteristics in relationship to COPD triage were identified through a multi-tier process. First, a comprehensive literature review of common institutional practices, published guidelines, and COPD assessment tests [32–35], clinical predictors and prediction models of exacerbations [25, 29, 36, 37], and current COPD management applications [38–41] was carried out. Once selected, the features were put under consideration by a panel of three board certified pulmonologists and one critical care specialist. This panel scrutinized and modified the variable list based on consensus practice methodologies and clinical experience. Finally, the questions, responses, and measures of each variable were generated and reviewed for content, conciseness, and patient appropriate language.

The question and response list from the aforementioned process defines a space of possible patient cases. To create the optimum set of data for training and validation, a statistical experimental design using the R optFederov package from the AlgDesign library was used. Each feature was modeled linearly. This method was applied to the profile variables and baseline vital signs to generate a diverse test set of 100 patient types. Once generated, the remaining symptom, current vital sign, and comorbidity features for each patient case were randomly selected in a Monte Carlo simulation based on known distributions and correlations in the literature [42–45] to create realistic patient scenarios.

The test set was shuffled and sent to a group of 6 pulmonologists to separately triage and assess the likelihood of an exacerbation. This set gave the physicians an opportunity to better assess the suite of patient health variables and provide feedback on the appropriateness of question language, completeness of clinical features, and realism of cases while actively triaging cases. The feedback from physicians was used to update the algorithm feature list and redesign a larger set of 2501 patient scenarios replete with baseline, vitals, and symptom data. In total, 101 cases were randomly selected for validation and 2400 cases were used for training.

Each of the 6 pulmonologists provided exacerbation and triage data in the training and validation datasets. An additional 3 pulmonologists contributed labels to the validation set. In particular, they provided,

1. A 1-5 rating of the severity of baseline, symptom, and vital sign variables
2. An assessment of whether or not the patients current health indicated a COPD exacerbation with 0-100% confidence
3. A recommendation for the appropriate triage action to take with 0-100% confidence.

The triage categories from which the physicians could choose were,

1. **Ok**: No additional medical attention needed,
2. **Plan**: Continue normal treatment and check back in 1-2 days,
3. **Doc**: Call the doctor,
4. **ER**: Go to the emergency room.

**Fig 1:**
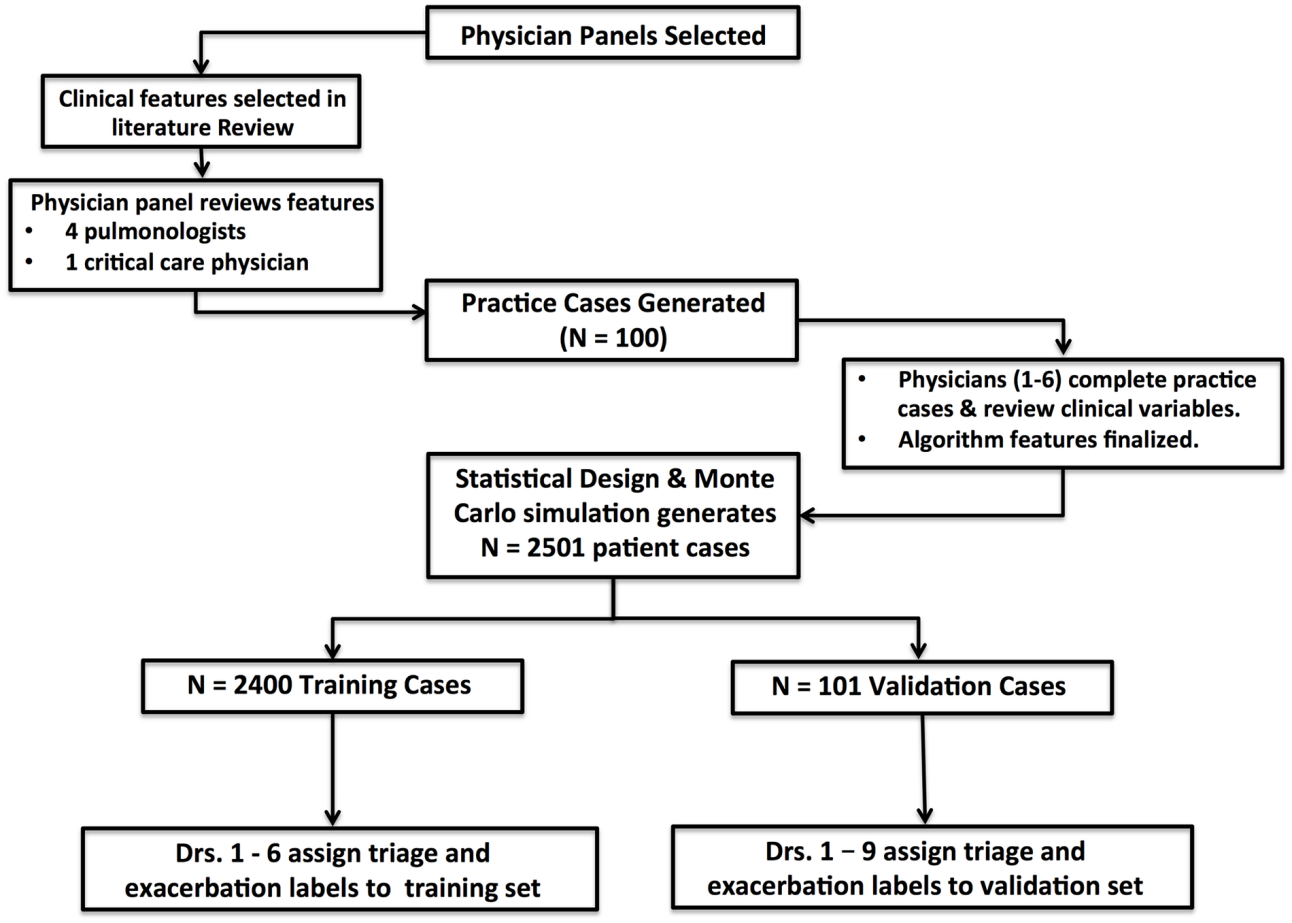
Process for generating patient case scenarios and collecting algorithm training and validation data.

Data was sent to physicians in 100-case batches. Triage and exacerbation assessments were recorded in spreadsheets akin to the sample shown in S1 Spreadsheet. Cases that were used in the training were individually labeled by physicians, while cases used in the validation set included the opinion of all 9 previously mentioned physicians. The process is depicted in Fig 1.

### Algorithm training and validation

**Fig 2:**
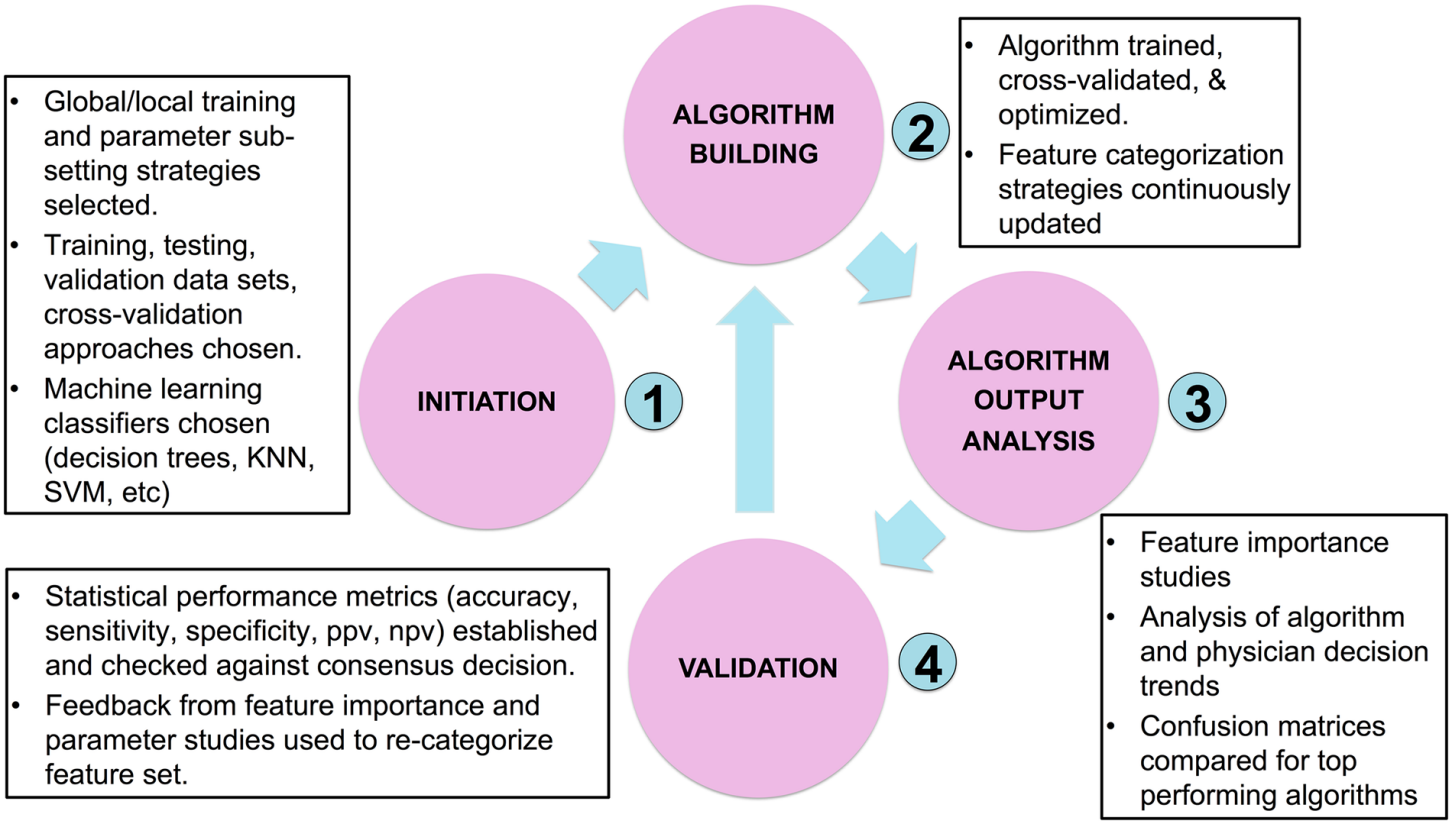
Training, validation, and optimization procedure for building COPD exacerbation and triage prediction algorithms.

The strategy used to find the optimal prediction model is shown in Fig 2. This process was identical both for predicting the presence of an exacerbation and predicting the appropriate triage recommendation. Initially, several candidate supervised learning classifiers were selected including support vector machines, logistic regression, Naive Bayes, KNN and a variety of gradient boosted and ensemble decision tree methods. For each classifier type, thousands of algorithms were trained on each combination of physician training data using Python’s Scikit-Learn suite. All algorithms went through a hyper-parameter optimization process including a grid search with 5-folds cross-validation. The top performing algorithms of each class were selected based on how they performed when making predictions on the out-of-sample validation test.

Algorithm predictions were validated by comparing the algorithm’s triage and exacerbation (y/n) classifications to the consensus decision of a panel of physicians on 101 hypothetical patient cases. Each individual physician and the algorithm were tested for how often their particular recommendation for a patient case matched the majority opinion. In cases of ties, the more conservative medical decision (higher triage/exacerbation category) was accepted as the correct one. The performance of the algorithm was compared to the other physicians in three scenarios: 1) The algorithm voted in the majority opinion, 2) The algorithm did not vote in the majority opinion, and 3) Neither the algorithm nor any individual physician voted in the majority opinion. The 101 validation cases were removed from the 2501 case set prior to training, which made them statistically diverse, clinically relevant, and truly out-of-sample. Statistical measures of performance used in this study included:

$$\text{Classification}\;\text{Accuracy}(\%)=\text{ACC}=\frac{TC}{TC+FC},$$ (1)

$$\text{Sensitivity}=\text{TPR}=\frac{TP_{ER}}{TP_{ER}+FN_{ER}},$$ (2)

$$\text{Specificity}=\text{TNR}=\frac{TN_{ER}}{TN_{ER}+FP_{ER}},$$ (3)

$$\text{Positive}\;\text{Predictive}\;\text{Value}=\text{PPV}=\frac{TP_{ER}}{TP_{ER}+FP_{ER}},$$ (4)

$$\text{Negative}\;\text{Predictive}\;\text{Value}=\text{NPV}=\frac{TN_{ER}}{TN_{ER}+FN_{ER}},$$ (5)

$$\text{Confusion}\;\text{Matrix}\;\text{Proximity}\;\text{to}\;\text{Upper}\;\text{Triangular}=\text{UTP}=1-\frac{LT}{101},$$ (6)

$$\text{Misclassifications}\;\text{Greater}\;\text{Than}\;\text{One}\;\text{Category}={\text{E}}_{\text{G}1}=\frac{C_{G1}}{101},$$ (7)

where,

1. *TC* = Total classifications matching consensus
2. *FC* = Total classifications not matching consensus
3. *TP*_*ER*_ = Emergency classifications matching consensus
4. *TN*_*ER*_ = Non-emergency classifications matching
5. *FP*_*ER*_ = Emergency classifications not matching consensus
6. *FN*_*ER*_ = Non-emergency classifications not matching consensus
7. *LT* = Number of lower triangle entries in confusion matrix
8. *C*_*G*1_ = Number of triage missclassifications greater than 1 category

Additional analysis was done to assess the algorithm’s performance on identifying situations in which “medical attention” was required. In particular, medical attention is defined as triage circumstances which call for physician assistance (triage category = 3) or emergency care (triage category = 4). The remaining two triage categories define instances in which no medical attention is needed. The statistical measures of performance for this study included:

$${\text{ACC}}_{\text{M}}=\frac{TC_{M}}{TC_{M}+FC_{M}},$$ (8)

$${\text{TPR}}_{\text{M}}=\frac{TP_{M}}{TP_{M}+FN_{M}},$$ (9)

$${\text{TNR}}_{\text{M}}=\frac{TN_{M}}{TN_{M}+FP_{M}},$$ (10)

$${\text{PPV}}_{\text{M}}=\frac{TP_{M}}{TP_{M}+FP_{M}},$$ (11)

$${\text{NPV}}_{\text{M}}=\frac{TN_{M}}{TN_{M}+FN_{M}},$$ (12)

where,

1. *TC*_*M*_ = Total classifications matching the consensus view on the need or lack of need for medical attention
2. *FC*_*M*_ = Total classifications not matching the consensus view on the need or lack of need for medical attention
3. *TP*_*M*_ = Sum of category 3 and 4 classifications made on cases with a consensus triage of at least category 3
4. *TN*_*M*_ = Sum of category 1 and 2 classifications made on cases with a consensus triage of at most category 2
5. *FP*_*M*_ = Sum of category 3 and 4 classifications made on cases with a consensus triage of at most category 2
6. *FN*_*M*_ = Sum of category 1 and 2 classifications made on cases with a consensus triage of at least category 3

Confusion matrices were used in this study to visualize the extent of algorithm and physician agreement with consensus for each triage class. Perfect confusion matrices are diagonal, indicating complete agreement between the triager and the consensus. Off-diagonal entries below the diagonal indicate under-triage with comparison to consensus while entries above the diagonal indicate over-triage. Subsequent result sections show such results.

### Algorithm feature importance

The importance of clinical variables in machine-learning predictions is calculated based on the methodology used by the particular model. In this study, the feature ranking of the Gradient-Boosted Decision Trees (GB) classifier was determined by the expected fraction of samples (case outcomes) to which a particular feature contributed across all trees in the ensemble. Higher fractions indicate higher feature importance. Ultimately, the fractional contribution was determined as an average across the entire forest.

In the case of Logistic Regression, the feature importance was determined by the size of the coefficient effect. When predicting triage, the Logistic Regression included a separate prediction model for each class relative to the other classes, conforming to one-vs-rest methodology. Hence, the feature importance was determined as the average rank of each feature effect over the four prediction models.

### Robustness of validation set consensus

The size and scope of the physician panel used in the validation set was a topic of great importance in this study. In order to assess the robustness of the consensus on the validation set, we started by selecting a minimum number of doctors (five) from the complete validation panel of 9 doctors + algorithm. After finding the majority triage opinion of the 5 physician panel on each case, we added a 6th doctor and calculated the percent of 101 total cases that changed triage labels. This process was repeated for all other physicians not in the 5-member panel and the results were averaged. Finally, the outcome of this procedure was averaged for every possible initial combination of 5 physicians to yield the average, max, and min percentage of cases where the majority decision changed after adding a 6th physician panel member. Using this method for all initial panel sizes generates a quantitative assessment of how many physicians are needed to establish a robust consensus in the validation set.

## Results

### Algorithm feature set

The clinical variables selected for algorithm training were found through the multi-tier process described in the method’s section, *Algorithm Feature Selection & Patient Case Generation*. The final variable list is shown in Table 1, and includes 1) patient background characteristics that are associated with COPD exacerbation risk and severity, 2) current clinical symptoms that encompass widely accepted features of exacerbations, and 3) physiologic measurements that are predicted to influence physician perception of exacerbation severity.

**Table 1:** List of patient profile, comorbidity, vital sign, and symptom factors, with respective measures, used in the COPD triage and exacerbation algorithms.

Variable		Units—Type
**Patient Profile**	Age	years—continuous
	Weight	lb—continuous
	Height	feet + inches—continuous
	Gender	Male/Female—categorical
	COPD GOLD STAGE	1,2,3,4—categorical
	Baseline MMRC Dyspnea	1,2,3,4,5—categorical
	Recent Exacerbations & Hospitalizations	Yes/No—categorical
	Lives Alone?	Yes/No—categorical
	Smoker	Yes/No—categorical
	Long-Term Oxygen User	Yes/No—categorical
	Assisted Daily Activity	Yes/No—categorical
**Comorbidities**	Congestive Heart Failure	Yes/No—categorical
	High Blood Pressure	Yes/No—categorical
	Coronary Artery Disease	Yes/No—categorical
	Diabetes	Yes/No—categorical
	Anemia	Yes/No—categorical
	Pulmonary Hypertension	Yes/No—categorical
	Acid Reflux	Yes/No—categorical
**Symptoms**	Shortness of Breath	1,2,3—categorical
	Cough	1,2,3—categorical
	Wheezing	1,2,3—categorical
	Change in Sputum Color	Yes/No—categorical
	Increased Sputum Volume	Yes/No—categorical
	Cold/URI	Yes?NO—categorical
	Medication Compliance	1,2,3—categorical
	Sleeplessness	Yes/No—categorical
	Current MMRC Dyspnea	1,2,3,4,5—categorical
**Vital Signs**	Oxygen Saturation	%—continuous
	FEV1	Vol/sec—continuous
	Heart Rate	BPM—continuous
	Temperature	°F—continuous

As the feature list included both continuous and categorical variables with different units and responses, the detailed questions and responses are included in S1 Document. The numerical levels of each categorical variable correspond to patient level responses. For example, in the case of *cough*, the levels 1,2,3 correspond to *less than usual, same as usual, and more than usual* respectively. All features have an additional response of *unknown* except for age, weight, height, gender, baseline dyspnea, and symptom questions. This was done to train the algorithm on cases in which patient data could be missing.

### Top performing algorithms

**Fig 3:**
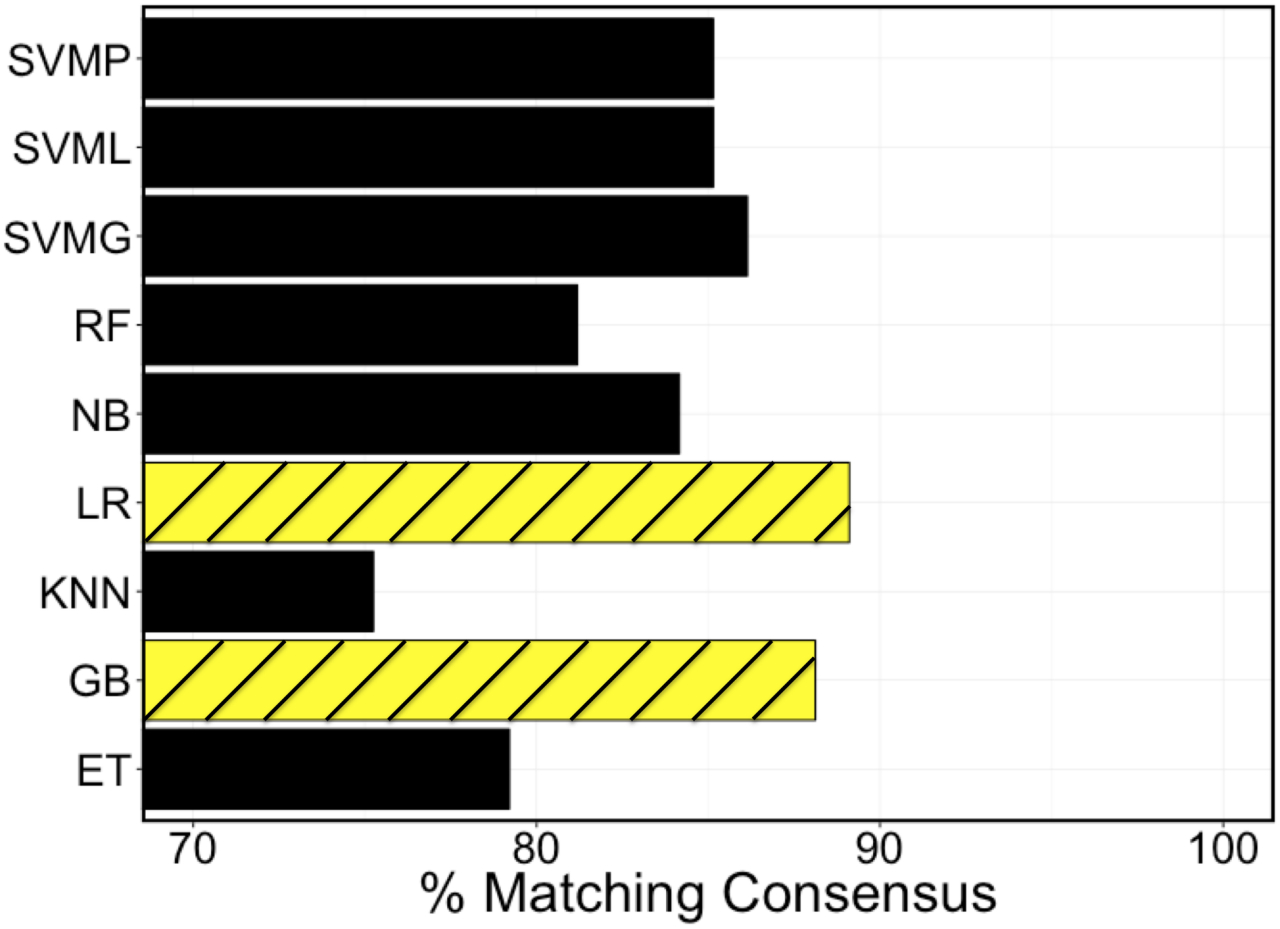
Comparison between ML classifiers at matching consensus decision in the validation set. SVMP, SVML, and SVMG are all support vector machine algorithms with polynomial, linear, and Gaussian kernels respectively. RF = Random Forest, NB = Naïve Bayes, LR = Logistic Regression, KNN = K-Nearest Neighbors, GB = Gradient Boosted Random Forest, and ET = Extra Decision Tree Classifier.

As detailed in the methods, patient cases generated using the variables shown in Table 1 were labeled by physicians, and the resultant data were used to train algorithms using a variety of strategies. Fig 3 includes a comparison of the top performing algorithms of each classifier type for out-of-sample classification accuracy. Among the different machine-learning classifiers tested, The top 2 performers were the Gradient-Boosted Decision Tree and the Logistic Regression. All classifier algorithm types were trained in a comparable way inclusive of hyper-parameter optimization and cross-validation.

### Algorithm performance

**Fig 4:**
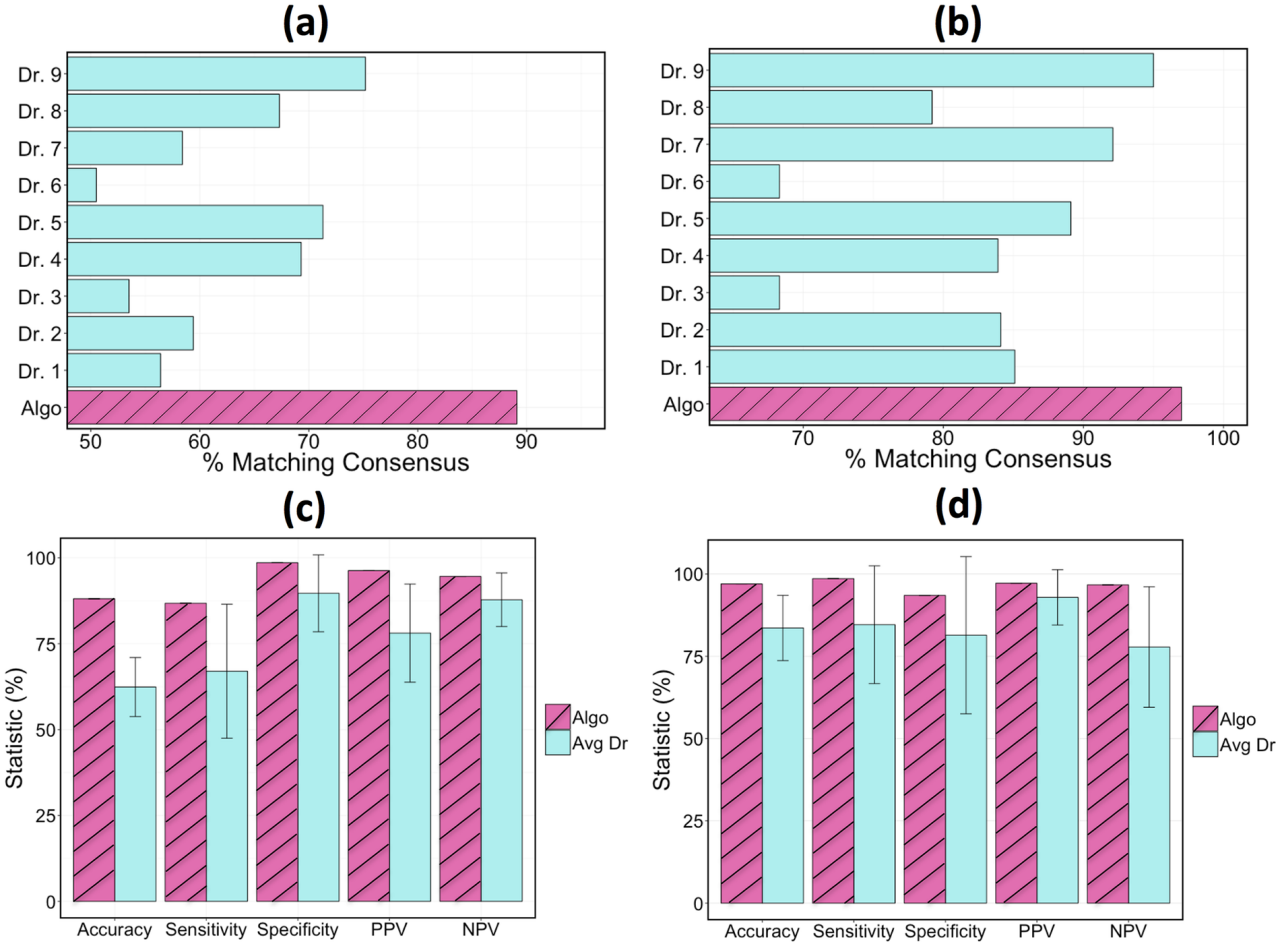
Performance comparison when the algorithm and all of the physicians got a vote in the consensus opinion. Comparison of the algorithm and individual physicians at predicting the consensus triage and exacerbation (y/n) in the validation set: (a) triage identification, (b) exacerbation identification. A comparison of the algorithm with the average physician in accuracy, sensitivity, specificity, ppv, and npv for: (c) triage identification, (d) exacerbation identification. Triage statistics were computed as defined in Eqs 1–7.

Model accuracy was measured as the percentage of classifications that matched the consensus triage and exacerbation labels in the validation set. The accuracy results of the GB classifier when the classifier voted in the consensus are depicted in Fig 4. The algorithm agreed with the consensus opinion in 88% of triage cases, whereas an individual physician agreed with the consensus 74% of the time at best. When determining if an exacerbation had occurred, the algorithm assessment again agreed with the consensus determination more than any individual doctor with a success rate of 97% as compared to 95% from the top performing physician. A comparison of the algorithm to the average physician performance is also shown in Fig 4. In the case of triage accuracy, sensitivity, and ppv, the algorithm performed more than 1 standard deviation better than the average physician (more than 2 standard deviations in the case of accuracy). The exhaustive set of statistical performance metrics for the top algorithms and the top physician are shown in Table 2.

**Table 2:** Statistical measures (Eqs 1–7) of triage and exacerbation identification ability for the top 2 performing algorithms and top physician.

Metric	Triage			Exacerbation		
	GB	LR	Top Dr.	GB	LR	Top Dr.
**ACC**	88.1	89.1	74.3	97.0	97.0	95.0
**TPR**	86.7	90.3	70.0	100	98.6	97.1
**TNR**	98.6	98.6	100	90.6	93.5	90.3
**PPV**	96.3	96.6	100	95.8	97.2	95.8
**NPV**	94.6	95.8	88.7	100	96.7	93.3
**UTP**	96.0	95.0	77.0	0.0	99.0	98.0
**E_G1_**	0.0	0.0	1.0	NA	NA	NA

**Fig 5:**
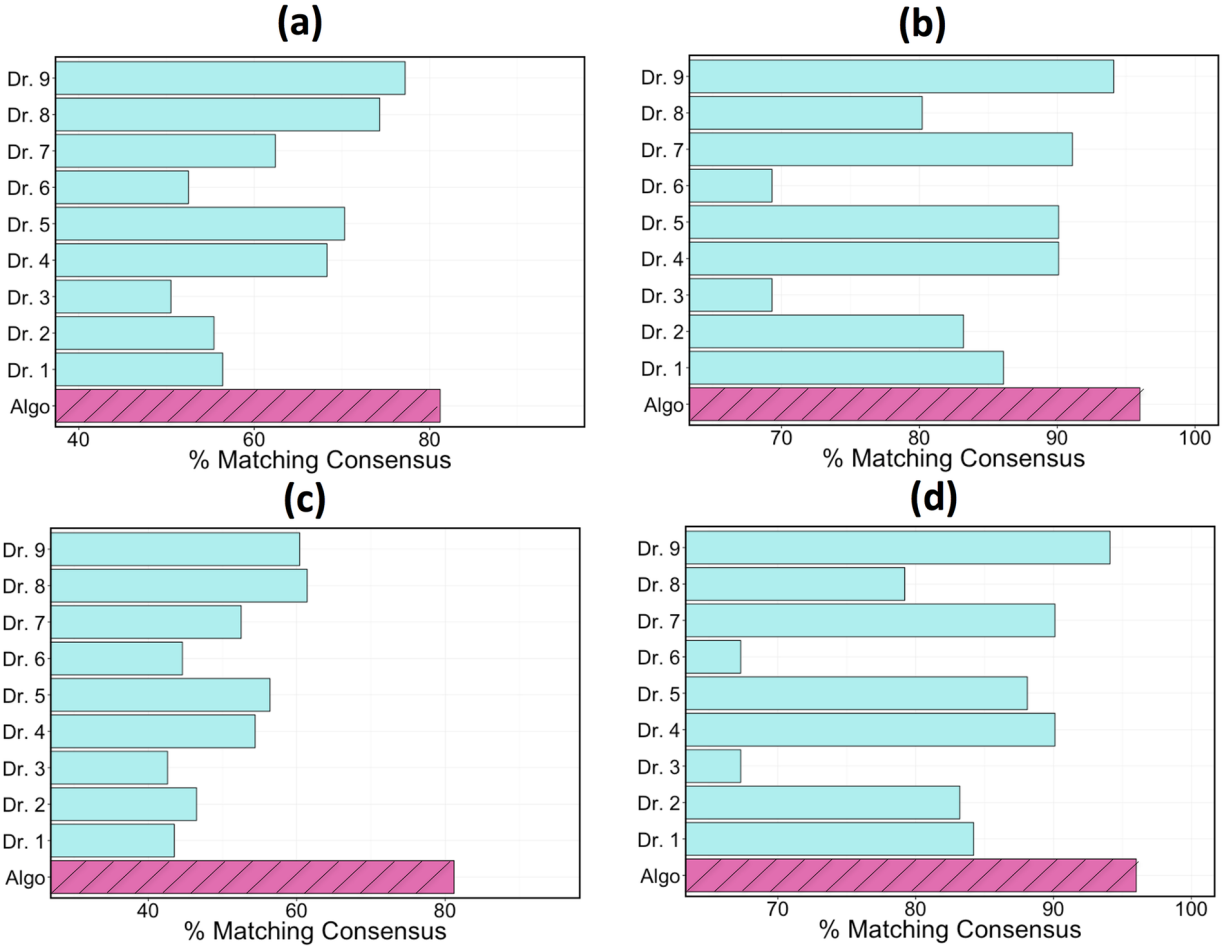
Performance comparison of algorithm and individual physicians at predicting the consensus of the validation sets. (a) triage performance, algorithm was not included in consensus, (b) exacerbation performance, algorithm was not included in consensus. (c) triage performance, no member votes when assessing their accuracy, (d) exacerbation performance, no member votes when assessing their accuracy.

It is noteworthy that the algorithm maintained its classification performance relative to the other physicians even in the assessments where it did not vote in the consensus. Results of these tests are shown in Fig 5. When the consensus opinion did not include the algorithm but included all individual physicians (a test that inherently favors the physicians), the algorithm had a triage/exacerbation classification accuracy of 82%/96% compared to the top performing physician at 77%/94%. In the case where no member’s vote was included in the consensus when calculating that member’s accuracy, the top physician dropped considerably in performance with triage and exacerbation accuracies of 62% and 93% respectively.

### Confusion matrix analysis

**Fig 6:**
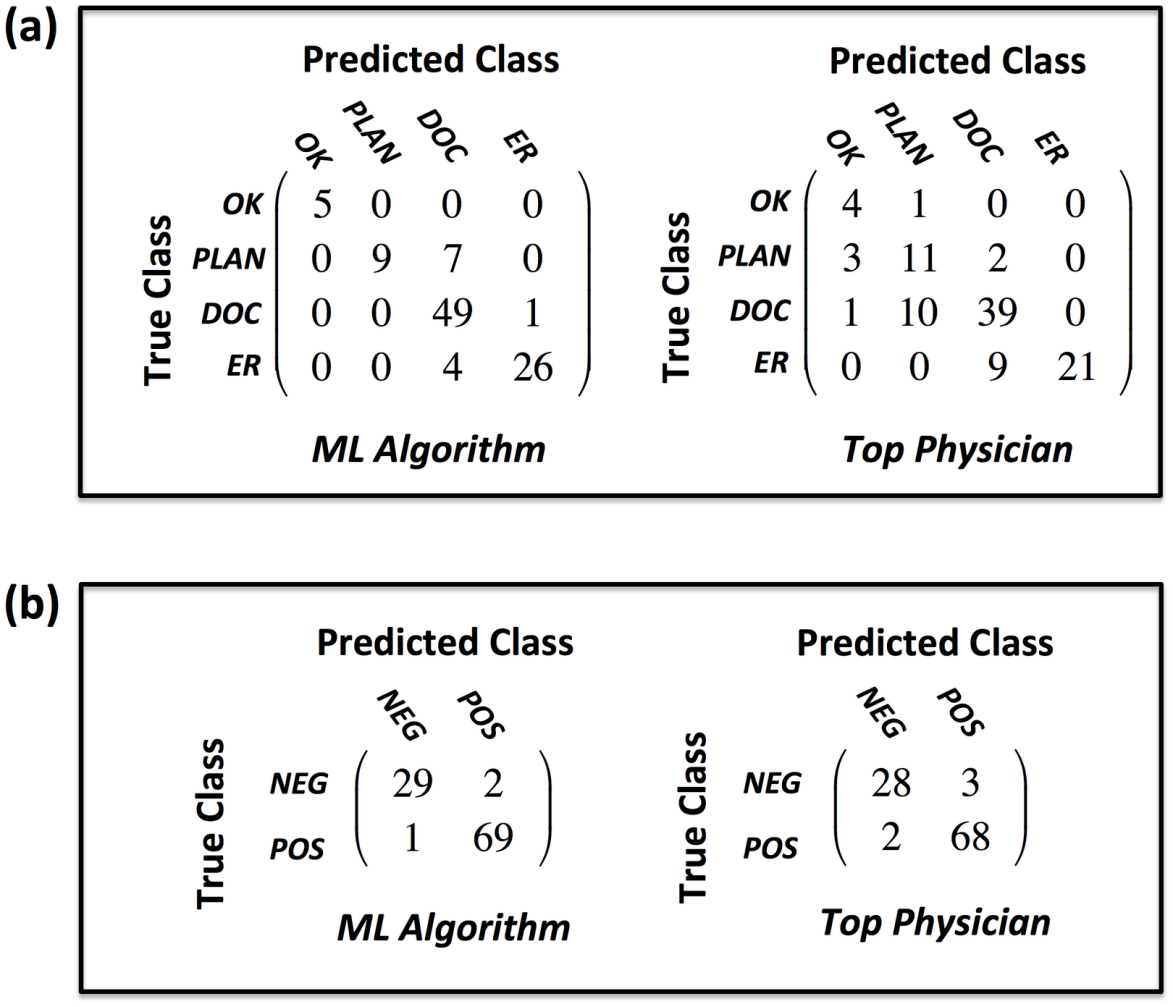
Confusion matrices comparing assessment performance of the GB algorithm to the top physician. (a) triage, (b) exacerbation. Note: top physician = the physician with highest classification accuracy.

The confusion matrices shown in Fig 6 give a comprehensive performance summary of both the algorithm and the top-performing physician (the physician with the highest classification accuracy on the validation set) in triage and exacerbation identification. On exacerbation identification, the top performing algorithm and top performing physician showed comparable performance when compared to consensus. On triage category identification, however, a number of performance and safety differences were observed

1. Performance observations
  - The algorithm showed a total classification accuracy of 88% while the top performing physician’s accuracy was 74%
  - The algorithm showed a sensitivity of 95% in triaging patients to the doctor and 87% in triaging patients to the emergency room. The top physician, by contrast, had a sensitivity of 78% and 70% respectively.
2. Safety Analysis
  - The algorithm never under or over triaged a patient by more than one category while the top physician under triaged one patient with *No additional medical attention needed* when the consensus triage was *Call your doctor*.
  - The algorithm never under triaged a patient who should be sent to the doctor while the top physician under triaged 22% of such patients. In ten out of eleven of those cases, the physician suggested that the patient consult their normal treatment plan and check back in 1-2 days and in one case that No additional medical attention is needed.
  - Out of 101 total cases, the algorithm under triaged the consensus less than 4% of the time. The top physician, by comparison, under triaged approximately 23% of the time.
  - For patients who have a consensus triage of *Go to the ER*, the physician under triaged 30% of them to the doctor while the algorithm under triaged less than 14% of them to the doctor.
  - When the algorithm didn’t agree with the consensus category 2 triage, it always triaged the patient to the doctor.
  - In comparison to the perfect confusion matrix the algorithm had 12 off-diagonal entries (88% accuracy) with 4 below the diagonal indicating that the algorithm under-triaged with respect to consensus 4% of the time and under-triaged when misclassifying 33% of the time. The top performing pulmonologist, by contrast, had 26 off-diagonal entries (74% accuracy) with 23 of those below the diagonal indicating that the top physician under-triaged with respect to consensus 23% of the time and under-triaged when misclassifying 88% of the time.

**Fig 7:**
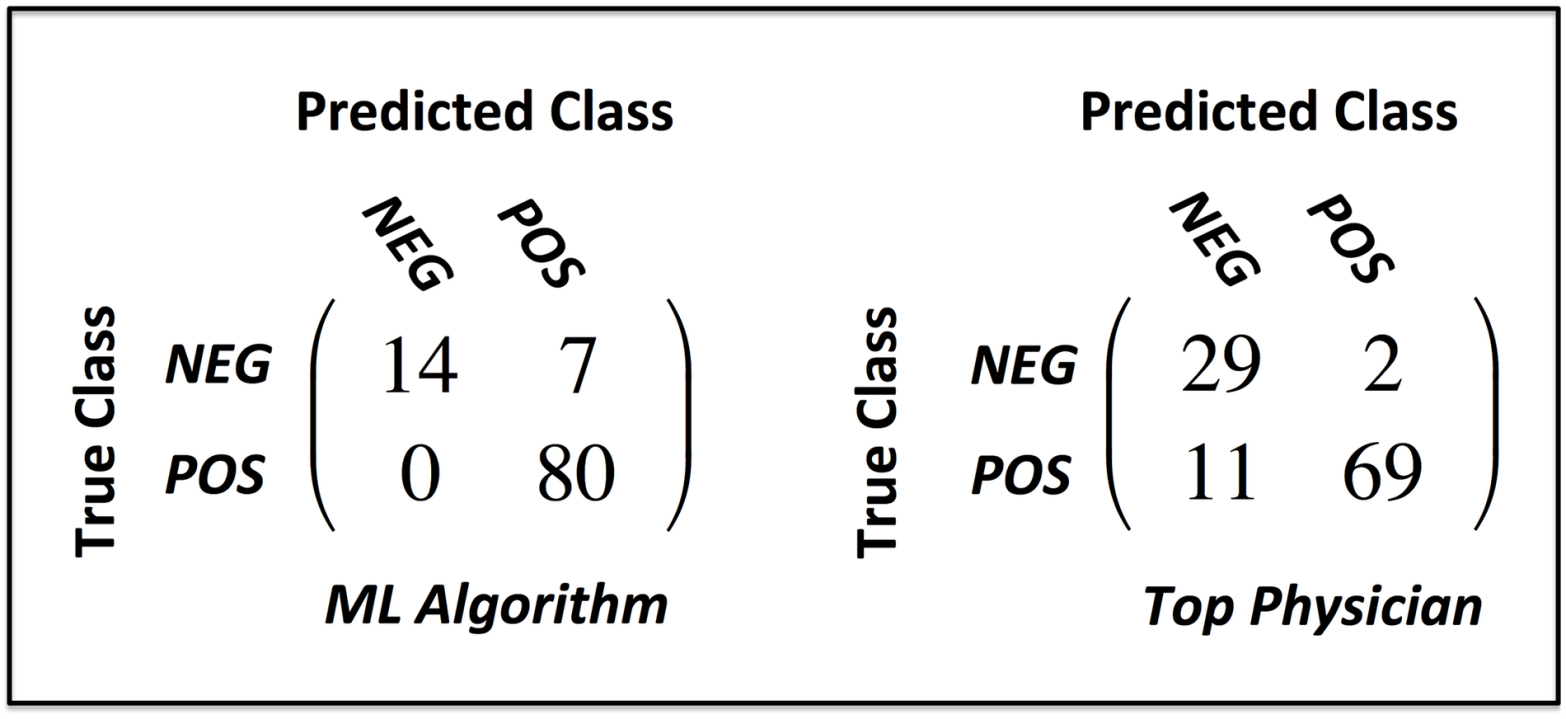
Comparing the performance of the GB algorithm to the top physician in assessing the need for medical attention. Note: top physician = physician with highest classification accuracy.

In the final study of algorithm performance, the algorithm and physicians were examined for their ability to discern the presence of general medical need (i.e: triage category of “Call your doctor” or “Go to the ER”). Statistical performance metrics of this study are given in Table 3 with confusion matrices shown in Fig 7. Similar observations can be made about the algorithms effectiveness though the top physician did exhibit superior performance in specificity and PPV over both algorithms. This is likely explained by the fact that the algorithm tended to over-triage in cases when it disagreed with consensus. It is further noteworthy that the algorithm never failed to identify the need for medical attention in the 101 validation cases, while the top performing physician misclassified 11 out of 80 such consensus instances (13.75% of the time).

**Table 3:** Statistical measures of performance of the top 2 algorithms (highest classification accuracy) and top performing physician when classifying the need for medical attention.

Metric	GB	LR	Top Dr.
**ACC**_**M**_	93.1	93.1	87.1
**TPR**_**M**_	100	97.5	86.3
**TNR**_**M**_	66.7	77.3	90.5
**PPV**_**M**_	92.0	93.9	97.2
**NPV**_**M**_	95.6	95.8	88.8

### Physician decision making trends

**Fig 8:**
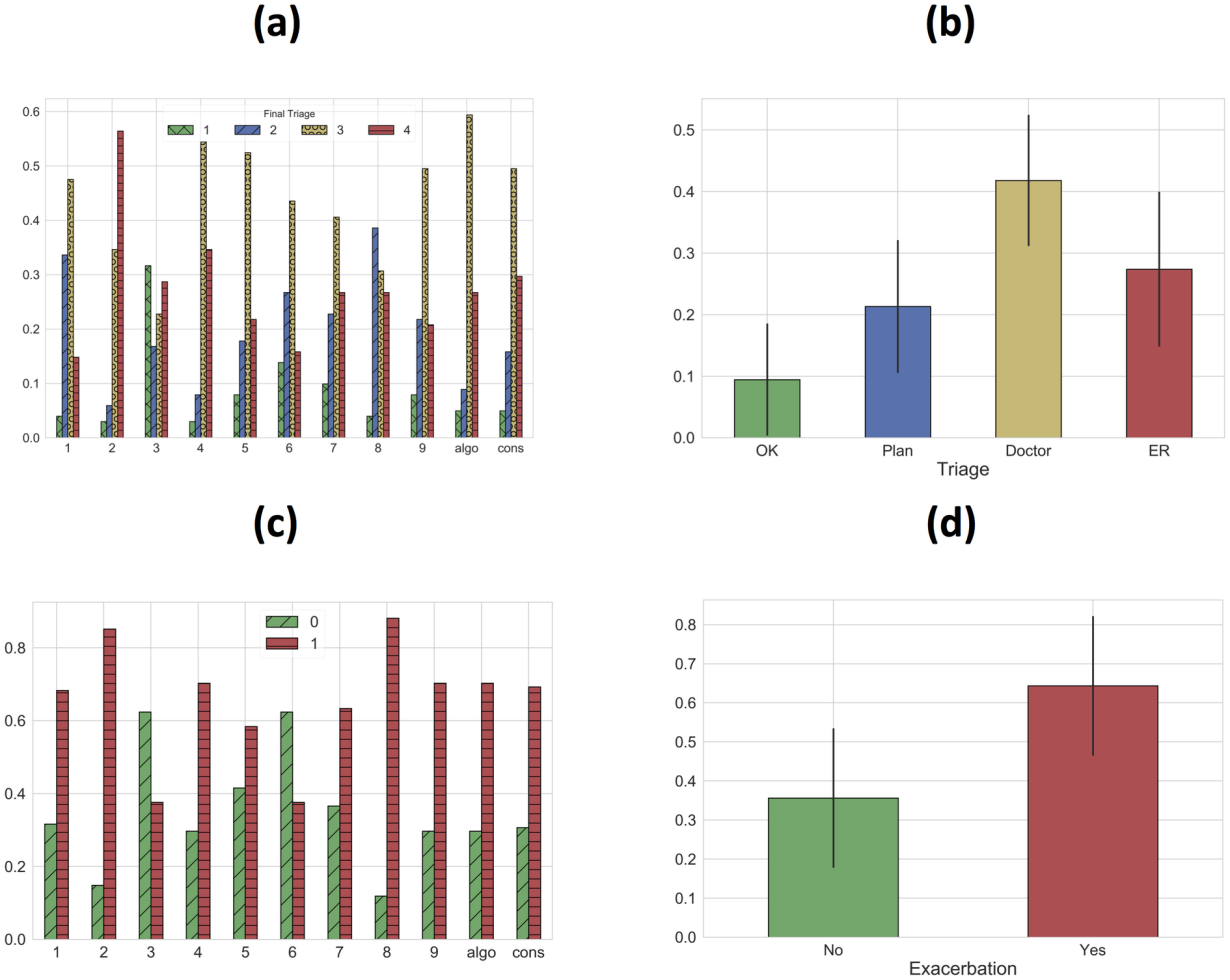
Distributions for each physician in the validation set (left) and the averaged distributions (right). (a) triage distribution, (b) averaged triage distribution, (c) exacerbation distribution, (d) averaged exacerbation distribution. Note: error bars indicate 1 standard deviation about the mean.

Physician-labeled data on exacerbation and triage categories were compared in the validation sets to better understand physician decision making. Fig 8 below shows the distribution of triage and exacerbation labels in the validation set per doctor. Plots of the average triage and exacerbation classes are also shown with error bars indicating 1 standard deviation intervals.

A variety of observations can be made about outlier opinions, inter-physician consistency, physician treatment of risk, and correlation between triage and exacerbation categories. Doctor 2, for example, triaged 63% of patients to the emergency room and Doctor 3 triaged 32% of patients as needing no additional medical attention, which are both over 2 standard deviations outside of the respective means. Physician triage assessment was also often highly independent of exacerbation assessment. Drs. 3 and 6, for example, had nearly identical exacerbation class distributions, but Dr. 3 triaged 32% of patients as needing no additional medical attention as opposed to 11% for that of Dr. 6. This could be partially due to a belief that an alternate diagnosis was driving symptoms. Moreover, the shape of the consensus triage distribution was matched closely only by the algorithm plus Drs. 5, 7, and 9. This suggests that the remaining physicians used a qualitatively different logic when choosing triage categories.

### Robustness of validation set consensus

**Fig 9:**
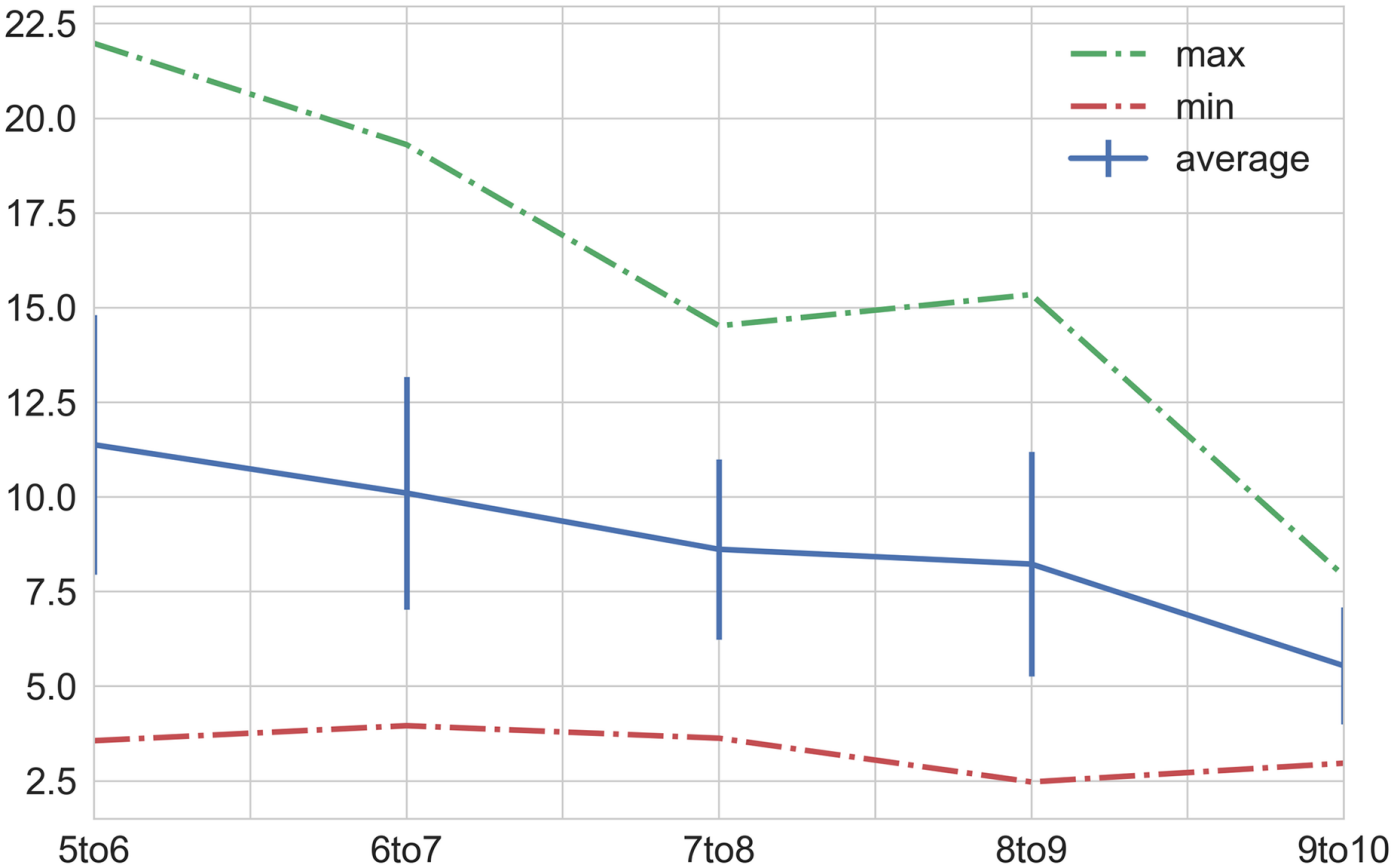
Plot of % change in consensus triage answers as additional doctors are added to the validation panel (plus algo). The average change when the panel reaches 10 members (from 9) is 5.5%.

The study of how many physicians constituted a robust validation panel (detailed in the Methods section) resulted in the convergence plot shown in Fig 9. Looking at the graph one can notice that each case of the validation set converged to an unchanging correct answer as more doctors were added. 7 physicians marked the region where the set showed good convergence with only 8% of cases changing on average when adding another doctor.

### Machine-learning feature importance

The patient variables that most influence triage and exacerbation assessment are shown in Table 4. Table 4 shows the hierarchical importance of the top 15 features in predicting the correct triage category based on the process described in the methods section *Algorithm Feature Importance*. Interestingly, the GB algorithm favored patient profile characteristics like age, BMI, and height as the most influential factors for triage while the same variables failed to reach the top 15 list for LR triage. LR also tended to weight vital sign levels more heavily when predicting on both triage and exacerbation classes. Despite these differences, both algorithms maintained comparably high statistical measures of performance.

**Table 4:** Feature importance for the top two performing algorithms for both triage and exacerbation models.

Triage		Exacerbation	
GB	LR	GB	LR
Age	O_2_Sat_(87, 89]	cough_3	cough_3
BMI	cough_3	sputum(col+vol)_0	shortbreath_3
Height	sputum(col+vol)_0	shortbreath_3	sputum(col+vol)_0
O_2_Sat_(87, 89]	shortbreath_3	wheeze_3	wheeze_3
shortbreath_3	wheeze_3	sputum(col+vol)_1	sputum(col+vol)_1
Infection	Sleeplessness	Δfev1_(-15, -5]	Δfev_(0, 10]
Sleeplessness	Infection	Δfev1_(0, 10]	med_comp_3
cough_3	hr_(100, 110]	O_2_Sat_(91, 93]	cough_1
wheeze_3	fev1_(0, 20]	sputum_vol_1	Δfev1_(-15, -5]
fev1_(0, 20]	O_2_Sat_(85, 87]	fev1_(90, 100]	O_2_Sat_(87, 89]
sputum(col+vol)_0	O_2_Sat_(0, 85]	med_comp_3	Sleeplessness
hr_(100, 110]	Δfev1_(-100, -15]	Age	ΔO_2_Sat_[-4,-2)_base < 93
O_2_Sat_(85, 87]	hr_(110, 120]	shortbreath_1	O_2_Sat_(85, 87]
heartrate_(110, 120]	sputum(col+vol)_1	Δfev_(-100, -15]	sputum_col_1
cough_1	hr>120	Sleeplessness	Δfev_(-5, 0]

## Discussion

The machine-learned triage approach in this study performed favorably when compared to individual physicians in a broad range of statistical performance measures both in triage and in predicting the presence of a COPD exacerbation. Unlike existing paper checklist type tools, the models incorporated the baseline medical health of the patient in a way that robustly accounted for the complex interactions of patient health variables. Gradient-Boosted Decision Trees and Logistic Regression showed the highest performance when making out-of-sample predictions on the validation set. The performance metrics used to evaluate the algorithms demonstrated accuracy, safety, consistency, and edge case prediction performance comparable to or better than the top performing physicians in all studies with three different assessments of consensus.

### Strengths

The use of machine-learning predictions on a clinical feature set to identify disease flare-ups and provide subsequent patient decision support is a unique contribution of this study. To date, we are not aware of any other work that has produced a comparable result. The use of consensus physician opinion as a validation standard and the analysis of individual physician performance on that standard is also a unique contribution of this study.

The current study has demonstrated that the top two performing algorithms, GB and LR, both yield a suite of statistical performance metrics that compare favorably with individual physicians, and yet, the clinical variables that most influence each model’s output maintain a different rank order of importance. Logistic regression generally weighted vital sign data with more importance. This result suggests that a good recommendation based on the validation standard in this study can be achieved through different logic (modeling type) with diverse health data (algorithm features).

### Limitations

While the algorithm exhibits very strong performance when predicting on the out-of-sample validation set, ultimately the algorithm training is done on cases with individual physician labels. Training on cases with more opinions could facilitate a more robust in-sample cross-validation process. Given the data collection methodology used in this study, it would become increasingly expensive and intractable to collect orders of magnitude more cases for training, but with increased access to electronic medical records, one could consider using a much larger patient dataset with historic patient outcomes guiding the training process. This approach would require considerable thought and further investigation given the lack of gold standard on what constitutes a correct triage assessment.

Thus far, the algorithm has been both developed and tested on hypothetical patient cases. Unlike clinical datasets for post-hoc analysis of hospitalized patients, data for outpatient triage and evaluation is not readily available, necessitating the use of simulated data. Although the feature set within the training data is certainly comprehensive and large when compared to the information that would generally be available to a pulmonologist, internist, or nurse in the clinical setting, an additional level of validation would be to compare the prediction of the algorithm in a real patient setting with a set of physicians actively triaging the same set of patients. This type of clinical data would provide additional insight based on current medical practices.

It is further recognized that the black-box nature of ensemble decision tree methods makes the decision making logic in triage recommendations difficult to interpret. The feature importance studies previously discussed shed light on which patient variables most influence the final outcome, but ultimately, the inherent complexity and interactions of the feature set make it difficult to give a simple, linear causal explanation of the algorithm output based on the inputted features.

### Future work

Mobile applications geared toward improved at-home patient care and self-management of chronic illnesses have substantially grown in use due largely to the availability of technology and the rising costs of health care [46]. While the growing popularity of mhealth (health care and public health practice supported by mobile devices [47]) is evident, its impact and efficacy is not [46]. This study has shown that machine-learning based applications offer the exciting prospect of accurate and personalized triage of COPD patients. Early detection of disease flare-ups and accurate council to patients has the potential to both reduce the severity of exacerbations and prevent unnecessary hospitalizations for otherwise healthy, anxious patients. This may assist the drive towards personalized medicine by better guiding decision support for individual patients.

The current algorithm is deployed in a mobile app that is primarily meant for at-home patient use, though it could also be used by nurses and internists less familiar with a patient’s baseline health as a tool to confirm their assessments during patient calls. The app should be further explored for its effectiveness in real patient populations with respect to various clinically relevant endpoints both for improving patient decision making and for engendering clinical reduction in severity and frequency of COPD exacerbations. Future investigation of the feasibility of machine-learning applications in clinical trials will be needed. Moreover, a robust clinical study on the influence of these applications on patient anxiety, stress, and overall health would elucidate.

With modern computational capability and continuously better access to health data, the opportunities to train machine-learning algorithms on large patient outcome datasets will improve. This may have particular relevance in COPD, where emerging data from large phenotyping and genotyping efforts, such as COPDGene and Spiromics, are delineating novel variables that impact exacerbation and disease risk [48]. Blood-based biomarkers and cellular content such as eosinophils, for example, are known to be correlated with increased risk of COPD exacerbations [49]. Although physician opinion is currently the gold standard for many clinical decisions, including diagnosis and triage of COPD exacerbations, active cloud-based training that integrates patient data in electronic medical records with available scientific knowledge may eventually provide specific predictions and recommendations that support medical-decision making. Such cloud-based information could be returned to a patient at home or to a provider in a clinical setting as APIs for computers and mobile devices.

## Conclusion

This study has shown that a machine-learning approach to triaging patients with COPD is a viable and robust method when compared to individual pulmonologists at facilitating at-home triage and exacerbation self-identification. The ML algorithm exhibited higher accuracy than all individual, board certified physicians in predicting the consensus opinion on both the presence of an exacerbation and the appropriate triage category in a representative set of patient cases. Furthermore, the algorithm erred in favor of patient safety more often than any individual pulmonologist and exhibited greater consistency in its recommendations. While the app is not meant to be a substitute for physician examinations or physician guided patient care, it does provide simple, easily accessible, safe, and highly accurate at-home decision support which can direct patients to the right care. Furthermore, it is generalizable to other chronic illnesses in which relevant symptom, signs, and patient profile data are available.

## Supporting information

S1 Table List of physicians, their affiliations and their contribution to the smart COPD development process. Pulmonologists who participated in this study provided expert opinion in clinical selection/review, algorithm training, and validation. The physician profiles are indicated in the supporting table.
(XLSX)

S1 Document Detailed questions and responses. (DOCX)

S1 Spreadsheet Training data sample. Simulated patient cases are issued to physicians to provide exacerbation and triage labels for the purpose of algorithm training and validation. This file includes a sample batch of 100 cases with the corresponding physician entered data.
(XLSX)

S1 Dataset Training and validation data. This file includes all the data used for training and validation.
(ZIP)
