# Supplementary material for: A machine learning approach to triaging patients with chronic obstructive pulmonary disease
Source: PLoS One. 2017 Nov 22;12(11):e0188532. doi: 10.1371/journal.pone.0188532 (PMC5699810; doi:10.1371/journal.pone.0188532)
Supplement: S1 Document — (DOCX) [file pone.0188532.s002.docx]

Algorithm Items & Responses

**Baseline**

1. **Tell us about yourself**
   1. Age
   2. Gender
   3. Height
   4. Weight
2. **Have you ever been diagnosed with (indicate all that apply)?**
   1. COPD
   2. Congestive Heart Failure
   3. Coronary Artery Disease
   4. Chronic Kidney Disease
   5. High Blood Pressure
   6. Pulmonary Hypertension
   7. Diabetes
   8. Acid Reflux
   9. Anemia
3. **Do any of the following apply to you (indicate all that apply)?**
   1. You live alone
   2. You use oxygen for your COPD
   3. You need help performing daily activities
   4. You were hospitalized for COPD in the last year
   5. You had two or more COPD flare-ups requiring antibiotics or steroids in the last year.
   6. You are a smoker
4. **What is your normal oxygen saturation level?**
5. **What is your normal heart rate?**
6. **What is your COPD Gold Stage/FEV1 Score?**
   1. Stage 4 - Very Severe (FEV1 < 30% normal)
   2. Stage 3 - Severe (FEV1 = 30-49% normal)
   3. Stage 2- Moderate (FEV1 = 50-79% normal)
   4. Stage 1 - Mild (FEV1 ≥ 80% normal)
   5. I don’t know
7. **Which statement best describes your usual breathlessness during activity?**
   1. Too breathless to leave the house and dress myself
   2. Need to stop to breath after walking about 100 yards
   3. Walk slower than people of my same age
   4. Breathless when hurrying on flat ground
   5. Breathless only with demanding exercise

------------------------

**Symptoms & Vitals:**

1. **What is your current oxygen saturation level?**
2. **What is your current FEV1?**
3. **Wat is your current heart rate?**
4. **What is your current temperature?**
5. **Have you had a recent worsening in any respiratory symptoms such as cough, shortness of breath, or waking up at night?**
   1. No
   2. Yes, for the last 24 hours
   3. Yes, for 1 to 3 days
   4. Yes, for more than 3 days
6. **Describe your shortness of breath?**
   1. Less than usual
   2. Same as usual
   3. More than usual
   4. More than usual AND I’m short of breath at rest.
7. **Which statement best describes your breathlessness during activity?**
   1. Breathless only with demanding exercise
   2. Breathless when hurrying on flat ground
   3. Walk slower than people of my same age
   4. Need to stop to breath after walking about 100 yards
   5. Too breathless to leave the house and dress myself
8. **Describe your cough?**
   1. Less than usual
   2. Same as usual
   3. More than usual
9. **Describe your wheezing?**
   1. Less than usual
   2. Same as usual
   3. More than usual
10. **Describe your change in sputum?**
    1. No change
    2. Change in sputum color
    3. Increased sputum volume
    4. Increased sputum volume AND change in sputum color
11. **Runny nose, sore throat, or feeling like you have a cold?**
    1. Yes
    2. No
12. **Are your respiratory symptoms waking you up at night more than usual?**
    1. Yes
    2. No
13. **How often are you taking your prescribed inhalers?**
    1. I don’t use prescription inhalers
    2. Less than usual
    3. Same as usual
    4. More than usual
